# The Feasibility of Shellac Wax Emulsion Oleogels as Low-Fat Spreads Analyzed by Means of Multidimensional Statistical Analysis

**DOI:** 10.3390/gels8110749

**Published:** 2022-11-18

**Authors:** Andreea Puşcaş, Vlad Mureşan

**Affiliations:** 1 Department of Food Engineering, Faculty of Food Science and Technology, University of Agricultural Sciences and Veterinary Medicine Cluj-Napoca, Calea Mănăştur Street, No. 3-5, 400372 Cluj-Napoca, Romania; 2 Technological Transfer Center “CTT-BioTech”, University of Agricultural Sciences and Veterinary Medicine Cluj-Napoca, Calea Floreşti Street, No. 64, 400509 Cluj-Napoca, Romania

**Keywords:** emulsion oleogels, shellac wax, three level two factorial design, cluster analysis

## Abstract

Shellac wax-based oleogel emulsions were studied with a three level two factorial design in order to find an optimal formulation for a spread formulation. Rheological, textural, colorimetry, and stability analysis were conducted to assess the performance of oleogel emulsions. FTIR spectra were also compared. The similarities between the samples were studied using cluster analysis. Analysis of variance (ANOVA) demonstrates that (i) the texture is influenced by the wax concentration, (ii) the rheology and stability by both the considered numeric factors (wax and water concentration) and their interaction, and (iii) the color by both factors. The emulsions containing 7% (m/m) shellac oleogels behaved like the strongest systems, (G′ & G_LVR_ > 30,000 Pa) and exhibited the highest value of the G′-G″ cross-over. The lowest oil binding capacity (OBC) was 99.88% for the sample with 3% (m/m) shellac and 20% (m/m) water. The whiteness index (Windex) varied between 58.12 and 78.50. The optimization process indicated that a formulation based on 4.29% (m/m) shellac wax and 24.13% (m/m) water was suitable as a low-fat spread.

## 1. Introduction

Giving the reported limitations of both oleogels and composite gel usage as fat-replacing systems in some foods such as bakery [1], meat [2], or dairy products [3], due to their less acceptable textural, sensory or stability properties and the possibility of designing foods with an even lower fat content by the means of oleogels emulsions formation, the study of the later systems is currently a research topic of interest with application in food [4]. It has been stated that some oleogelators exhibit interfacial properties and can be used for developing more stable Oleogel-in-Water (Og/W) or W/Og emulsions than the conventional ones [5].

The optimization of both oleogels and oleogel-based emulsions was proposed very recently by different approaches such as multi-response optimization using a desirability function [6] or surface response methodology [7,8]. Tekiki et al. studied the formulation of new spreads based on olive oil and honey, stabilized by bees wax, using a Box Behnken design, as a response for the increasing demand of the low-fat food market and to enrich the knowledge related to the structural behavior of these products [9]. Emulgels were also studied by a Quality Design approach for future pharmaceutical and biomedical applications [10]. Previous studies were aiming to study processing conditions by means of multi objective optimization in order to obtain Myverol 18–50 XL PL (composed of 89.35% stearic acid, 3.86% palmitic acid, 3.22% saturated fatty acids, 2.13% glycerol, and 1.45% diglycerides)-based oleogels similar to margarines in terms of textural, rheological and oil binding properties [6].

Some oleogel emulsions that are formed with sitosterol:oryzanol mixtures as structuring agents were reported to be unstable since they are sensitive towards water [11]. In the case of waxes, it was revealed that their molecular composition influence the stability of oleogel emulsions; candelilla wax, which is mainly formed by n-alkanes, can cause destabilization, while sunflower and rice bran wax, which are composed of wax esters, allow the formation of stable emulsions that can be compared to spreads [12]. Due to the low stability of the resulting emulsions, hybrid systems consisting of wax and also a food grade emulsifier were mostly explored as agents for W/Og systems [4]. The influence of different water contents on the properties and structure of emulsion oleogels structured with a wax–starch mixture was also recently explored by Wang, Liqian, et al., who presented some technical references and a theoretical basis that is important for expanding the so-called low-oil oleogel industry [13].

Shellac wax has already been used solely for formulating oleogel emulsions that were stable due to their complex composition, with alkanes, esters, fatty alcohols, and acids of the wax, and thus both polar and non-polar ingredients; however, the already developed emulsions contained a low water amount (20%) [14]. Shellac exhibited an activity at the interface of the water and oil and decreased the tension, but also crystallized during cooling in the bulk oil, forming a microstructure that entrapped the water droplets [14]. Given its good structure formation and high stability, the applicability of shellac oleogels and even shellac wax oleogel emulsions, but with only 25% water content, in foods such as sponge cake, margarines, or chocolate has already been explored [15]. Developing shellac wax-based emulsion oleogels with high water content (40%) might be used for designing food products such as meat, dairy, or bakery products with an even lower fat content and improved fatty acid profile.

A hot simultaneous oleogelation and emulsification approach was proposed by Öğütcü et al. in the development of breakfast margarine composed of a virgin olive oil-beeswax oleogel emulsion (with or without the addition of emulsifiers and hydrocolloids), and some formulation were stable in terms of texture, color, and oxidation for up to 90 days [16]. However, again relatively low water content was used in the formulation of these emulsions (10%, 20%, or 25%), since some commercially available emulsions require up to 40% water phase.

The dispersion of cocoa and icing sugar in an oil medium leads to the formation of a soft solid (chocolate paste) which does not present phase separation when oil binders such as palm fat or hydrogenated oils are used. A more recent study demonstrated that a cocoa spread free of palm fat can be formulated with an emulsion composed of milk fat and a cellulose ether [17,18].

Among the several factors affecting the performance of oleogel emulsions, the most relevant are: the concentration of structuring agents, their properties, such as their nature or molecular weight (which will influence their solubility in the dispersed mediums), the water content, and the presence of other emulsifies [4]. The stability of oleogel emulsions after formation seems to be affected by the particles playing the emulsifier role due to their crystallization in bulk oil and re-organization after homogenization. The stability of oleogel in water emulsions (Og/W) has also been studied in different mediums (with temperature or pH variations) [19].

In the present study, the oleogel emulsion formation is based on a three-level two-factorial design (3^2^); one of the aims of the study was to assess which factor (water or wax concentration) influences the properties of the oleogel emulsions, and to acquire response functions. Modelation of the curvature of a specific response can be possible by introducing a chosen concentration of wax or water in the equations computed by the Design expert software. Using three-level designs, the quadratic relationship between the response and each of the factors can be revealed.

Secondly, numerical optimization was performed to predict the optimal formulation of the emulsion oleogel with similar properties to commercially available spreads. The similarity levels between the emulsions and a low-fat margarine alternative were also assessed. To further understand the composition of the shellac wax based emulsionated oleogels, FTIR spectra were acquired and analyzed.

## 2. Results and Discussion

### 2.1. Response Analysis

Oleogels were prepared at three different concentrations of shellac wax, this being considered one of the numeric factors of the design: 3% (m/m) shellac wax represented the low level (−1), 5% (m/m) the mid level (0), and 7% (m/m) the high level (1). The water concentration employed for the manufacturing of the oleogel emulsions was the other numeric factor.

A design matrix was generated by the software and the responses were introduced based on the results from the textural, rheological, stability, colorimetry analysis conducted on the samples, as seen in Table 1.

#### 2.1.1. Textural Analysis

Hardness and adhesiveness were analyzed as main textural attributes relevant for developing spreads. The hardness of the emulsions varied between 54–600 g, while the adhesiveness between 27–255 g. Other spreads containing only 20% olive oil and 80% water stabilized by beeswax, with the inclusion of honey in the continuous phase, presented higher values of hardness (between 95.5 and 184.9 N)–the variation being also due to the stirring time and speed applied during manufacturing. In the current study, the ratio of the minimal and maximal values of the considered textural parameters are close to 10 (11.11 and 9.44, respectively) and no transformation of the data was suggested by the software to model the responses. For the analyzed textural parameters, the quadratic, two factor interaction (2FI), and linear models are proposed as being relevant by the Design expert software, however, in each case, the predicted R^2^ was not as close to the adjusted R^2^, so the data transformation was applied to improve the modelling.

For the hardness, the Natural log transformation was applied to the responses, and the ANOVA analysis reveals that F (12.55) and *p* (0.0318) values are significant (p-95%) for a chosen quadratic model. The lack of fit was not significant, which was desired because we wanted our model to fit. High values for R^2^ were obtained (0.95) and the Adeq Precision was 10.15, suggesting the model can be used to navigate the design space. The equation in terms of actual factors that can be used to make predictions about the hardness for given levels of each factors given by the DesignExpert software was the following:

ln(Hardness)= −0.7467 + (water% × 0.1177) + (structurant × 1.1956) − (0.0028 × water% × structurants%) − (0.0012 × water%^2^) − (0.0605 × structurant%^2^). (1)

**Figure 1:**
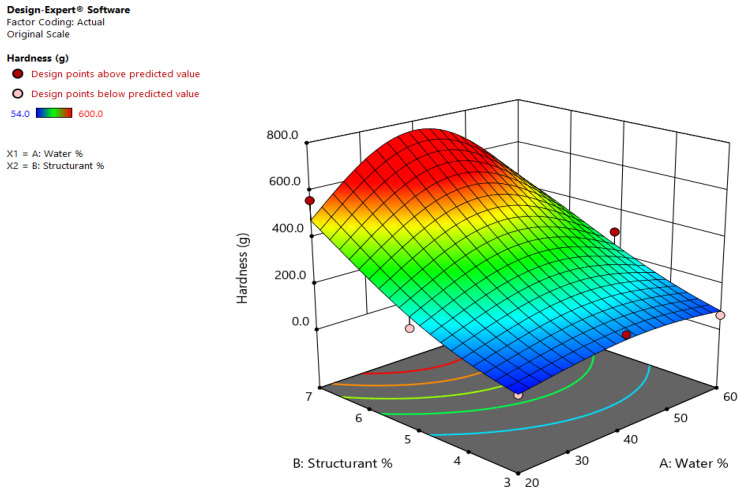
Effects of wax and water concentration on the hardness of shellac wax oleogel emulsions.

The model suggests that the hardness values are significantly influenced by the concentration of the wax (*p* = 0.005), while the water content does not significantly affect the texture of the emulsions, as seen in Figure 1. The highest value of hardness (600 g) was registered by EM3, which is formed with 40% (m/m) water and 7% (m/m) shellac wax oleogel, followed by the emulsions structured with the same amount of wax (7% (m/m)), but 20% (m/m) of water (560.5 g-EM4) or that with 60% (m/m) water (415.5 g–EM1). A high value of hardness was also registered for the sample containing 40% water and 5% (m/m) wax (342 g-EM7), indicating that the formulation of the emulsion with 40% (m/m) water will lead to well-structured and firm systems.

The textural parameters of oleogel emulsions consisting of virgin olive oil and beeswax with or without the addition of Tween20 or Tween80 as emulsifiers and an amount of 25% water, was assessed in the study by Öğütcü et al., given their importance in determining the spreadability of the composition [16]. The emulsion registered values of less than 100 g for hardness, while a breakfast margarine considered the control sample registered a hardness of 250 g which is less firm that the emulsions formulated in the present study [16]. Therefore, shellac wax-based oleogel emulsions, except for EM6 and EM8, might be suitable as margarine replacers in terms of texture.

The study of Toro Vasquez et al. also demonstrated that the crystal mass of the structuring agents (in their study candelilla wax and monoglyceride being used) influences the hardness of some oleogel emulsions [20]. The manufacturing of the samples was conducted in a high pressure homogenizer, which permits various pressure and temperature protocols [20].

For the adhesiveness, the Natural log transformation was also applied to the responses, and the ANOVA analysis reveals that F (12.11), *p* (0.0333) and the quadratic model are significant (p-95%). There is only a 3.33% chance that an F-value this large could occur due to noise. However, looking at the model, not all coefficients are significant (p-95%), as seen in Table 2, but these terms were kept in the model formation in order to support the hierarchy of the quadratic model.

**Figure 2:**
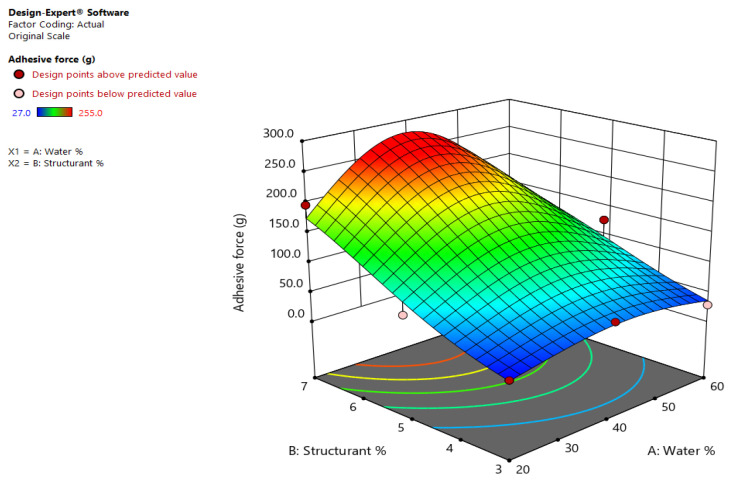
Effects of wax and water concentration on the adhesive force of shellac wax oleogel emulsions.

Despite the high value for R^2^ (0.9528), there was a large difference between the adjusted R^2^ and the predicted R^2^. However, precision measures the signal to noise ratio, and the Adeq precision was 10.13, which is higher than 4 and indicates an adequate signal [21]. This model can be used to navigate the design space. Again, the concentration of shellac wax dictates the curvature of the model function, the samples with the lowest adhesive force being those containing 3% (m/m) shellac wax oleogel, as seen in Figure 2.

The increase with the adhesiveness and the content of the structuring/stabilizing agent increases were also observed in the spreads developed by Tekiki et al. [9]. The resulting function that allows us to determine the adhesiveness based on the components of the emulsions is:

Ln (adhesive force) = −1.6851 + (0.1269 × Water%) + (1.2465 × Structurant%) − (0.003041 × Water% × Structurant%) − (0.001373 × Water%^2^) − (0.071051 × Structurant%^2^). (2)

#### 2.1.2. Rheological Behavior

The quadratic model was also employed in the study of the rheological properties (G_LVR_, G cross point) of the shellac wax emulsion oleogels. The models were statistically significant for each parameter, with a *p* of 0.0091 and 0.0226, respectively, and F values of 30.09 and 17.29, respectively. For the responses representing G_LVR,_ the natural log transformation was applied, while for the G cross over point the inverse square root. An R^2^ of 0.9804 and similar values for the predicted and adjusted R^2^ were obtained in case of G_LVR,_ as seen in Table 2. In order to make predictions with regard to possible values of G_LVR_ of oleogel emulsions having the compositional parameters within the minimum and maximum concentration set in this design, the following equation is proposed:

ln (G_LVR_) = +0.109631 + (0.189312 × Water%) + (1.60279 × Structurant%) − (0.009322 × Water% × Structurant%) − (0.001547 × water%^2^) − (0.059350 × structurants%^2^). (3)

**Figure 3:**
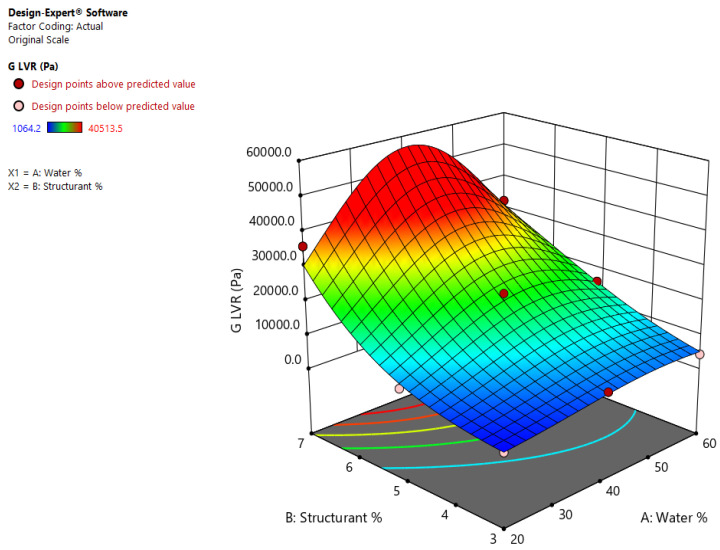
Effects of wax and water concentration on the determined G’_LVR_ of shellac wax oleogel emulsions.

The viscoelastic properties of shellac oleogel emulsions were statistically affected by both water and wax concentration and their interaction, as seen in Figure 3.

The emulsions containing 7% (m/m) shellac oleogels (EM1, EM3, EM4) behaved like the strongest gels, with G′ and G_LVR_ reaching values > 30,000 Pa. A gel-like behavior of the oleogel emulsions is previously reported as being due to the oleogelation of the oil phase [22]. Strong gel-like behavior was exhibited by emulsions of oleogel structured with 5% shellac wax, except for the sample containing 20% (m/m) water, which had a G_LVR_ of only 5946.3 Pa. This sample is similar to the emulsions containing oleogels structured with 3% (m/m) shellac wax (EM5, EM8), except for the sample with 20% (m/m) water (EM6). For these reasons, it seems that the inclusion of higher amounts of water is desired in order to improve the functionality of the emulsion oleogels in terms of rheological behavior. The rheological curves of the emulsion oleogels are presented as Supplementary Figure S1.

In the study of Patel et al., emulsions formed with 2 or 4% shellac wax and 20% water had a more liquid consistency, while the emulsion with 6% shellac wax had a solid-like behavior; both the loss and storage modulus reached 60,000 Pa, and the oleogels strength was found to be dependent on the shellac wax concentration [14].

**Figure 4:**
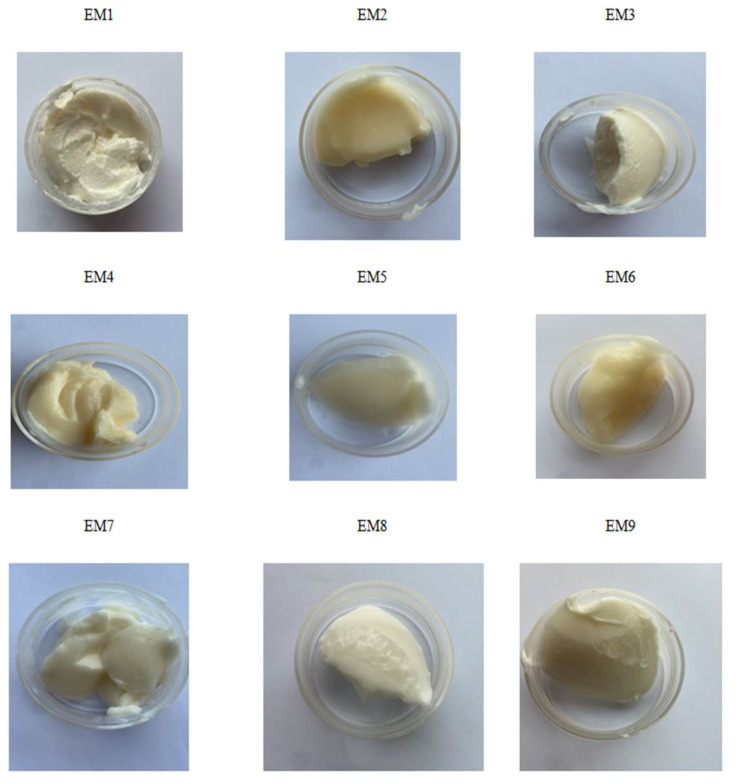
Appearance and macroscopic properties of the emulsions obtained from shellac wax-based oleogels.

In the current study, EM6 (consisting of 20% (m/m) water and oleogel structured with 3% (m/m) wax) registered the lowest values for G′ and G″ in the amplitude sweep test and a G_LVR_ of 1064.2 Pa, being the weakest system of all, but still exhibited after manufacturing solid-like behavior, as seen in Figure 4.

Interestingly, samples containing 3% (m/m) shellac wax and 40% (m/m) water (EM5) or 60% (m/m) water (EM8) exhibited stronger gel-like behavior, and a G_LVR_ of 5014.4 Pa and 4264.2 Pa, respectively, supporting the fact that water concentration also positively affects the rheology of the samples. However, the emulsification process and therefore water inclusion in the system reduces the strength of the microstructure in comparison to that of the initial oleogels, not because water will negatively influence the system, but because of the smaller crystalline amount contained by the emulsions in comparison to the oleogels, as it was previously reported by other authors [23,24].

The study of shellac oleogel emulsions was also employed for samples manufactured with 25% water in another the work of Patel et al., and strong gel-like systems were obtained, the complex modulus G* having values larger than 10,000 Pa for the sample containing 6% shellac wax and 25% water [14]. Sunflower wax-based oleogel emulsions containing 20% water exhibited G′ and G″ values higher than 10^3^ Pa in the amplitude sweep test [25]. It was then stated that higher amounts of water can be used in the formulation of wax-based emulsion oleogels, with no additional surfactants and strong gel-like systems being developed.

In the current study, the linear viscous region (LVR) of all the oleogel emulsions varied from 0.01% to 0.1%; a cross-over point could be determined for each sample–where G′ = G″, the strain was higher than 1%, and after that point the samples began to flow, losing their gel-like behavior.

A decrease in G′ and an increase in G″, the strain was higher were observed during the amplitude sweeps as the strain was increased, the point where their values cross being known as the G′-G″ cross-over point and possibly representing a measure of emulsion destabilization due to shear forces. The determined values ranged between 38–1731.5 Pa, and thus the transformation of the data was employed in order to model the response. A high R^2^ of 0.9638 was obtained, but high differences occurred between the predicted and adjusted R^2^, thus model reduction and the usage of a two-factor interaction model might improve the data modelling. However, the Adeq precision was of 17.29 and the quadratic model can be used to navigate the design space, the equation in terms of actual factors being the following:

1/Sqrt(Gcross) = +0.299127 − (0.002515 × Water%) − (0.031981 × Structurant%) + (0.000391 × Water% × Structurant%) + (3.49863 × 10^−6^ × Water%^2^) − 0.000890 × Structurant%^2^). (4)

**Figure 5:**
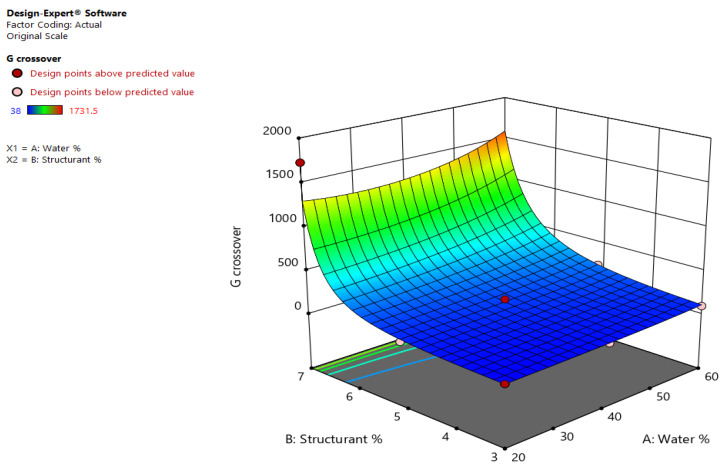
Effects of wax and water concentration on the determined G_crossover_ point of shellac wax oleogel emulsions.

The highest value of the G′-G″ cross-over point was registered for emulsions containing oleogel structured with 7% (m/m) shellac wax. In this case the G′-G″ cross-over occurred at 1028.28 Pa for EM1 (60% (m/m) water), at 462.44 Pa for EM3 (40% (m/m) water), and at 1731.5 Pa for EM4 (20% (m/m) water). Lower values for the cross-over point were registered for emulsions containing oleogels with a lower wax content; the next high cross-over value (223.84 Pa) was registered for sample EM9 (60% (m/m) water and 5% (m/m) shellac), thus the increase in water content and increased shellac amount lead to high G′-G″ cross-over points, as seen in Figure 5. A cross-over between G′ and G″ was also previously observed in the emulsion oleogels formed with rice bran wax and PGPR; for these samples, the determined values decreased with increasing water content [23].

This again demonstrated that water enforces the strength of the shellac wax oleogel emulsion network, and the increased concentration of water increases the rheological stability of the samples.

#### 2.1.3. Stability

Stability is an important issue of the emulsion oleogels and is also assessed by other authors in different conditions using different protocols including physical, thermal, and freeze-thawing stability analysis [19]. It was stated that in the case of the emulsion oleogels with high stability, the mobility of the droplets is decreased and a much firmer and cohesive system is developed [26]. The emulsions with the highest stability of 99.99% and 99.98% (EM1, EM3) registered high values of firmness (600 g and 415.5 g, respectively), but interestingly EM4 containing 20% (m/m) water and 7% (m/m) shellac wax oleogel, despite having a firm structure and a hardness value of 560.5 g, displayed a relatively lower stability (99.96%).

A less stable emulsion was EM5, which is formed by 40% (m/m) water and emulsion structured with 3% (m/m) shellac, because it exhibited a lower OBC (99.92%). The lowest oil-binding capacity (OBC) and stability values were 99.88% for the emulsion containing 3% (m/m) shellac but only 20% (m/m) water (EM6), and thus we can assume that the presence of higher water amounts increases the physical stability of the emulsions. The values of stability registered for shellac wax emulsion oleogel were higher than the values registered for oleogel emulsions prepared in other studies with varying amounts of sunflower wax, Tween 20, and Span 20 as emulsifiers, depending on the final composition, which had an OBC of 97–91% [25].

The quadratic model (*p* = 0.0011) employed for analyzing stability revealed high values for the predicted R^2^ (0.9954) and the adjusted R^2^ (0.9878). The following equation can be used for predicting the stability of different shellac wax-based oleogel emulsions:

ln (Stability) = +4.60248 + (0.000028 × Water%) + (0.000417 × Structurant%) − (2.50325 × 10^−6^ × Water% × Structurant%) − (4.17772 × 10^−8^ × Water%^2^) − (0.000017 × Structurant%^2^) (5)

**Figure 6:**
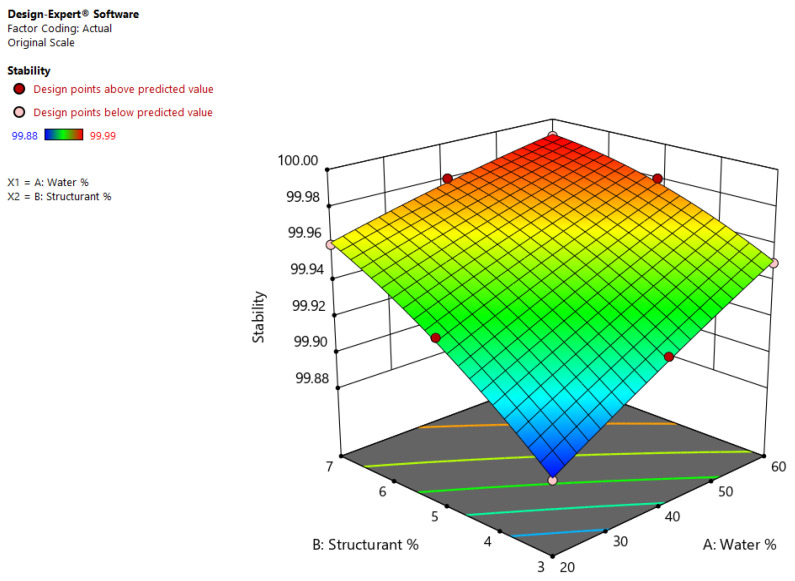
Effects of wax and water concentration on Stability of shellac wax-based oleogel emulsions.

The model strengthens the fact that stability is influenced by both wax and water concentration (*p* < 0.0001) and their interaction (*p* = 0.0075), as illustrated in Figure 6.

#### 2.1.4. Colorimetry

Color is an important parameter that is reported to be influenced by the water content of the emulsions or the quality of the oil, also influencing the sensory attributes that determine the acceptance and suitability for food products [27]. The Whiteness index, calculated from the instrumental color parameters of emulsion oleogels: a* (redness), b* (yellowness), L* (lightness), ranged from 58.12 to 78.50. The Natural Log transformation was applied in order to improve the model and to maximize the adjusted R^2^ and the predicted R^2^. The Predicted R^2^ of 0.6978 was not in reasonable agreement with the Adjusted R^2^ of 0.9207. The equation in terms of actual factors that can be used to make predictions about the response for given levels of each parameter, because of the Adeq precision which was 11.88, is as follows:

ln (Windex) = +3.82411 + (0.010549 × Water%) + (0.012602 × Structurant%) − (0.000017 × Water%) × (Structurant% − 0.000051 × Water%^2^) + (0.000347 × Structurant%^2^). (6)

**Figure 7:**
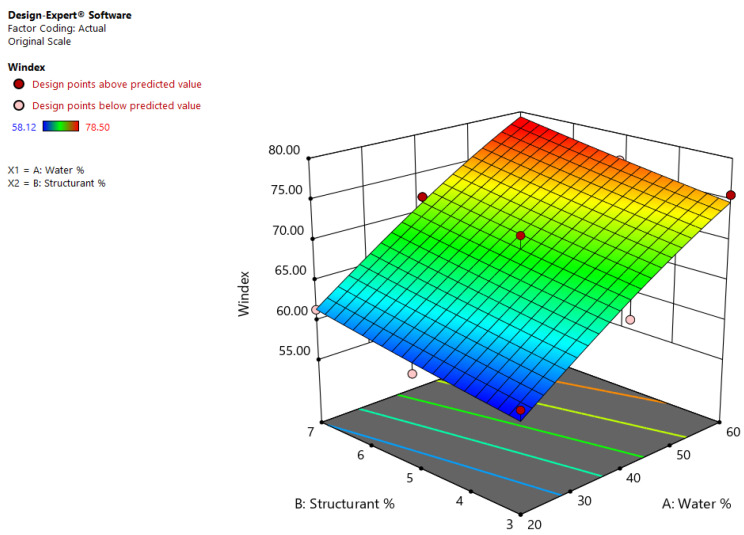
Effects of wax and water concentration on the calculated Whiteness index of shellac wax oleogel emulsions.

Even if other studies previously suggested that color parameters are influenced by all the ingredients used for developing a novel system [28], for the oleogel emulsions, Windex was significantly affected only by the water concentration, not by the factor interactions or their squares. The higher the water content in the emulsion formulation, the higher the value of Windex was, as seen in Figure 7. In addition, the lower the wax percentage in the emulsion formulation, the lower the Windex value was. EM2 and EM6, with 20% (m/m) water and 5% (m/m) and 3% (m/m) shellac wax, respectively, had a low Windex of 58.12 and 58.99.

### 2.2. Optimization of the Emulsion Composition Using Three Level Factorial Design

For this purpose, wax concentration was minimized and water concentration was maximized, while the rest of the responses were explored differently.

At first, hardness was maximized, adhesive force minimized; G_LVR_ was maximized, along with the G _cross over_ values, while the rest of the parameters were kept in range. All the parameters were assigned with the same importance coefficient. Overall, nine solutions were suggested by the Design expert software, with a desirability of 0.6405. An emulsion formed with 4.58% (m/m) shellac wax-based oleogel and 60% (m/m) water would meet the selected criteria.

Secondly, the adhesive force criteria was minimized, the G_LVR_ and Stability maximized, while the rest of the parameters were kept in range and 14 solutions were offered, with the highest desirability coefficient being 0.6689 for samples consisting of 60% (m/m) water and 4.39% (m/m) shellac wax.

**Figure 8:**
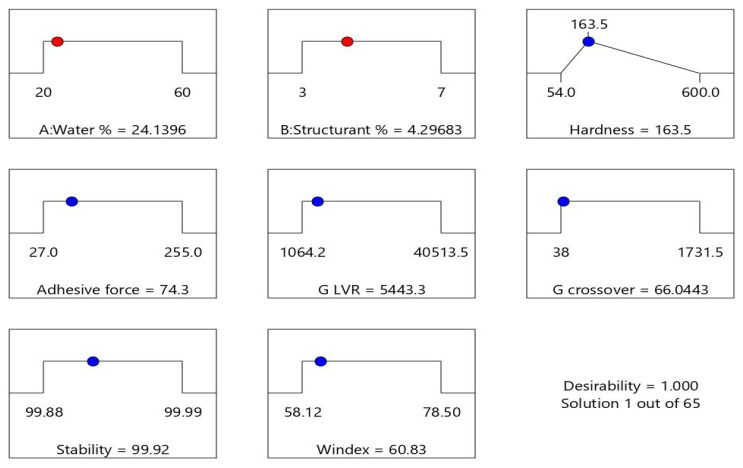
The calculated concentration (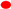) of water (A) and shellac wax (B) and the responses (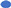) of shellac wax-based oleogel emulsion suitable as low-fat margarine replacer, as given by Design Expert Software in the optimization test.

The reference margarine presented a hardness of 163.5 g and, in order to determine whether shellac wax-based emulsion oleogels are suitable as margarine replacers, all the responses were kept in range–except for hardness, which targeted this value. A number of 65 solutions were given by the software, all with 1 as the highest desirability coefficient, and the numerical factors and the results of the responses being also given, as seen in Figure 8.

The software proposes an oleogel emulsion formulation with 24.13% (m/m) water and an oleogel structured with 4.29% (m/m) shellac wax, with the properties displayed by Figure 8. The convenient solution can be chosen based on the desired parameters of the responses.

### 2.3. Cluster Analysis

**Figure 9:**
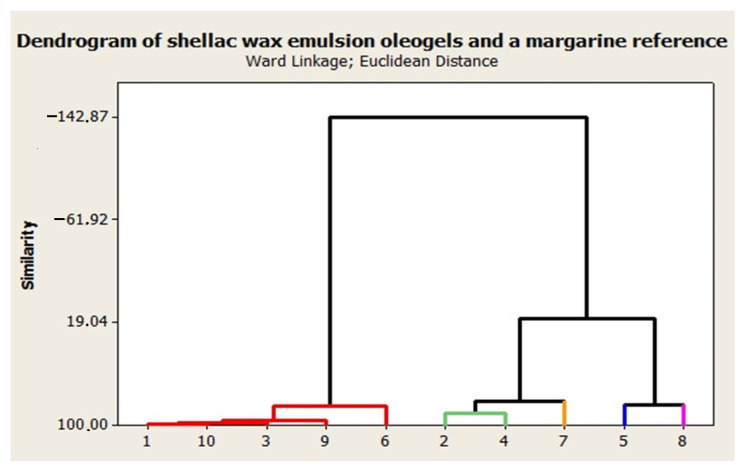
The dendrogram of shellac wax emulsion oleogels (1–9) and the margarine formulation (10), based on the structural analysis (Cluster 1 **–**, Cluster 2 **–**, Cluster 3 **–**, Cluster 4 **–**, Cluster 5 **–**)

The similarity levels between the emulsion and the formulation proposed in Section 2.2 as a low-fat margarine alternative can be visualized on the dendrogram represented in Figure 9. As one might expect, the cluster formation of the emulsions was based on the same water or wax content even if they were not considered as variables. The ward linkage method was used to keep the distance level as low as possible. The Euclidian distance measure was also selected.

When the formation of five clusters was selected, the similarity levels within the same clusters were high and varied between 84.75–96.19%. Cluster 1 included EM1 and EM3, which both have 7% (m/m) shellac wax oleogel in composition and 40–60% (m/m) water, but also EM9, which consists of 5% (m/m) shellac wax and 60% (m/m) water. The margarine proposed as reference was also included in Cluster 1 (as sample 10) along with EM6, which is made from 3% (m/m) shellac and 20% (m/m) water.

This clusterization confirms that spreads similar to fat-reduced margarines can be designed by including 40–60% (m/m) water in the formulation, because they present similar properties to those with lower water content (20–25%). Cluster 2 is represented by EM2 and EM4, which contain only 20% water and 5%, respectively, and 7% (m/m) shellac wax. This can be included in the same cluster as EM7 when the formation of three clusters is selected, but similarity levels are decreased to 81.30%. The emulsions containing oleogel structured with 3% (m/m) shellac wax and 40% (m/m) water alone form Cluster 4, but could be included in the same cluster as EM8, since they present a similarity level of 83.86%.

### 2.4. FTIR

**Figure 10:**
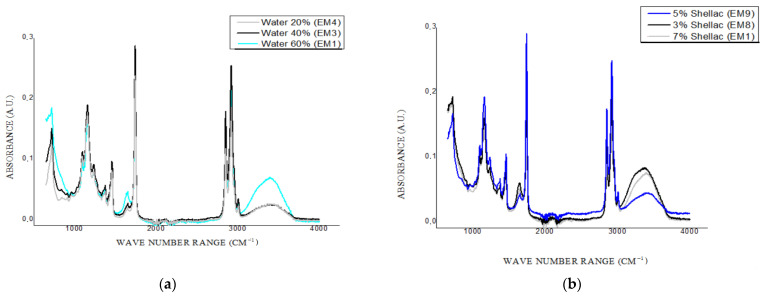

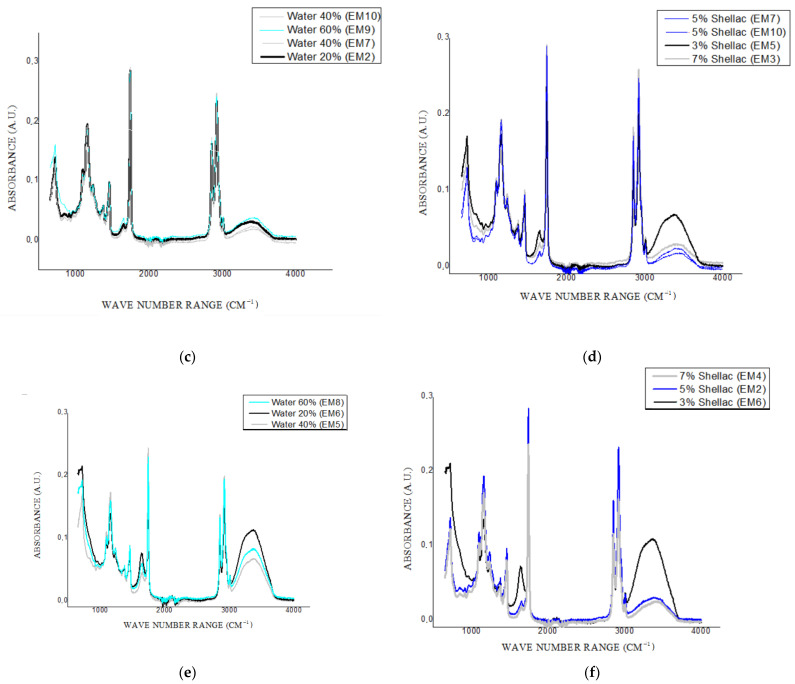
FTIR spectra of shellac wax-based emulsion oleogels, containing (**a**) 7% shellac wax and various water concentrations (20–60%), (**b**) 60% water and various shellac concentration (3–7%), (**c**) 5% shellac wax and various water concentration (20–60%), (**d**) 40% water and various shellac concentration (3–7%), (**e**) 3% Shellac wax and various water concentration (20–60%), and (**f**) 20% water and various shellac concentration (3–7%).

The FTIR spectra of the emulsions were displayed in Figure 10 in such a manner that allows us to evaluate the influence of water concentration or that of wax concentration on the oleogel emulsion structures. Following the previous reports, the registered peaks can be assigned to various functional groups. All the emulsion samples exhibited peaks between 3000–4000 cm^−1^ due to the O-H bonds of water. EM6 and EM2 exhibited a peak at 3360 cm^−1^ (Figure 10f), while EM8 and EM5 at 3377 cm^−1^ (Figure 10e), and the rest of the samples exhibited peaks even more shifted to the right–above 3410 cm^−1^. The conformational changes are highlighted by these shifts. Lower intensities of these peaks were registered by the emulsions containing 5% (m/m) wax-based oleogel in the formulation (EM2, EM7, EM9, and EM10) and those with 7% (m/m) wax-based oleogel, except for EM1, as seen in Figure 10a,c, respectively.

Peaks at 3009 cm^−1^ are specific for the stretching vibrations of cis alkene -HC=CH-of unsaturated fatty acids from sunflower oil and are exhibited by all of the emulsion samples, with variations in their absorbances. Peaks at 2916 cm^−1^ and 2848 cm^−1^ are reported to be a characteristic C-H vibration of waxes [29]. Regardless of the wax concentration, the emulsions with 40% (m/m) water registered peaks with the highest absorbance, following EM3 (7% wax) > EM10/EM7 (5% (m/m) wax) > EM5 (3% (m/m) wax).

At 1745 cm^−1^, the C-O stretch of functional groups contained by the lipids and fatty acids or the wax esters leads to the formation of peaks [29]. Both water and wax concentration influence the peaks occurring at 1640 cm^−1^, mainly due to O-H groups, and the highest absorbances were registered for samples containing 3% (m/m) shellac wax oleogel (EM5, EM6, EM8). Changes in the vibrational characteristics of the hydrocarbon chain of the shellac wax are exhibited at 1461 cm^−1^ due to the C-H bending of n-alkanes and at 719 cm^−1^ due to the CH_2_ rocking vibration, and can offer information about the role of the wax in the emulsification process [30,31]. High absorbances were registered for EM3 and EM4. Peak displayed by EM1 in this region registered similar intensities to that of oleogels containing 5% (m/m) shellac wax. Peaks at 1374 cm^−1^ are due to the presence of polyphenols originating from sunflower oil, while the peaks resulting in the wave-number interval of 1232–984 cm^−1^ highlight complex vibrational interactions due to both hydrocarbons and esters presents in the oil.

## 3. Conclusions

Low-caloric products or lower amounts of trans- or saturated fats can be designed by using emulsion oleogels in foods. High variability was registered for the structural, rheological, appearance, and stability parameters of W/Og systems designed with various concentrations of shellac wax (3–7% (m/m)) and water (20–60%(m/m)) based on a 3^2^-factorial design. By using RSM, the impact of the compositional factors and their interaction on the performance of the shellac wax-based oleogel emulsions was elucidated. The obtained quadratic models allow the prediction of the behavior of the emulsions formed from oleogels structured with shellac and might speed the usage as novel food applications. The current study demonstrates the functionality of shellac wax-based emulsion oleogels with a water content up to 40–60% (m/m) and similar properties to a margarine.

It is demonstrated that shellac wax permits the formulation of stable emulsions even when it is used in a concentration as low as 3% (m/m), with water contents up to 60% (m/m). In terms of the performed analysis, the less performant emulsion was EM6 and registered a hardness of 54 g, G_LVR_ of 1064.2 Pa, and a stability of 99.88%, but this is still similar to a margarine formulation, as demonstrated by the multivariate analysis. The inclusion of higher water contents (40–60% (m/m)) enforce the activity of shellac wax, increasing the textural, rheological, and stability performances of the oleogel emulsions. The emulsions containing higher wax percentages (5–7% (m/m)) displayed more prominent solid-like behavior and increased stability, being suitable even as shortenings or more plasticized fats.

An oleogel emulsion formed with 4.29% (m/m) shellac wax and 24.13% (m/m) water meets the criteria set in the optimization test in order to elaborate a fat-reduced margarine alternative. However, the formulation possibilities are more vast since the functionality of the product is met even by the emulsions containing 60% (m/m) water and 7% (m/m) wax (EM1), 40% (m/m) water and 7% (m/m) wax (EM3), or 60% (m/m) water and 5% (m/m) wax (EM9).

## 4. Materials and Methods

### 4.1. Materials

Sunflower oil was purchased from a local market (Cluj Napoca, Romania) and used without further processing. Shellac wax (7302L) was kindly donated by Kahl Wax (Trittau, Germany). Distilled water was used for the elaboration of the emulsions.

### 4.2. Oleogel and Emulsion Preparation

For preparing the oleogels, the oil and wax were weighted and heated on above the highest melting point of shellac wax (80 °C) on a magnetic hot stirrer plate IKA C-MAG HS 7 (Staufen, Germany) under mild stirring condition (300 rpm/min). Then, oleogel was mixed with water to prepare the emulsion oleogels (EM), using T25 IKA Ultra-turrax (Staufen, Germany) (15,000 rpm/min for 2 min). Water (at room temperature) was mixed with the oleogel (at 80 °C) with no additional emulsifier.

To prepare the emulsion oleogels, the miscellaneous response surface methodology of three level two factorial design was used in the Design Expert Sofware V8.0.7.1 (Stat-Ease Inc., Minneapolis, MN, USA). Emulsions, regardless of the water content, do not display destabilization and were self-standing when the upside-down test was performed.

Overall, nine treatment combinations, which are minimum for this type of design, dictate the emulsions’ composition (EM1–EM9). A three-level two-factorial design is convenient for the low number of samples that are necessary, in comparison to the central composite design that computes samples on five levels.

### 4.3. Textural Analysis

To assess the texture profile analysis (TPA) of the emulsions, a CT3 Brookfield Texture Analyzer (5 mm target distance, 1 mm/s test and post-test speed, trigger load 1 g) equipped with a 10 kg load cell and a cylindrical probe (TA25/1000; with a diameter of 25 mm) was used.

### 4.4. Rheological Response

The linear viscoelastic region (LVR) of the emulsions and the non-destructive deformation range was measured by small amplitude dynamic measurement using a rheometer Anton Paar MCR302, equipped with a parallel plate geometry (PP25, with a diameter of 25 mm), a peltier sytem, and Julabo waterbath. Dynamic stress sweep measurement was at a frequency of 1 Hz at 20 °C with oscillatory stress varying from 0.01 to 100%. Prior to measurement, emulsions were kept at room temperature for at least 2 h.

### 4.5. Stability

The centrifugal stability of the emulsions was determined according to Szymanska et al. with slight modifications, using centrifuge DLAB DM0412 (Rowland St. City of Industry, CA, USA) [32]. The samples (5 g) were centrifuged at 3000 rot min^−1^ for 30 min (at 23 ± 2 °C). After centrifugation, the tubes were inverted for 30 min to remove the released oil. The comparison and ratio of the mass of the sample before and after a centrifugation cycle allowed the assessment of emulsion stability.

### 4.6. Colorimetry

The color measurement of shellac wax-based emulsion oleogels was performed with the portable colorimeter NR200 (3NH, Shenzhen, China). For each emulsion, the Hunter Whiteness Index (Windex) was calculated using the lightness L*, a* (−a greenness, +a redness) and b* (−b blueness, +b yellowness) color parameters provided by the equipment, as following:

(7) $$\mathrm{Windex}=\sqrt{{\left(100-L^{*}\right)}^{2}+{a^{*}}^{2}+{b^{*}}^{2}}$$

### 4.7. Fourier Transform Infrared Spectroscopy

The FTIR spectra of the shellac wax-based emulsion oleogels were acquired in ATR mode with Agilent Cary 630 FTIR. Samples were taken directly (consecutively, one by one) from the fridge and placed on the FTIR. Background was collected and then spectra were scanned in the 600–4000 cm^−1^ wave number range with a resolution of 4 cm^−1^ and 64 scans, an adapted method from Öğütcü, M et al [16]. and Biswajit Sena et al. [33]. Spectra were analyzed with Origin PRO8 software.

### 4.8. Statistics

The output responses were performed at least in duplicate and expressed as means. The analysis of variance (ANOVA) was performed to validate the developed quadratic models for each analyzed response and to evaluate their fitness. The *p*-value, F-value, coefficient of determination (R2), and Adequate Precision were also analyzed. The developed models were statistically significant. The three-level designs facilitate the investigation of a quadratic relationship between the response and each of the factors; for these reasons, the quadratic models were employed and reported due to their significant *p* value and Adeq Precision.

The three-dimensional response surface graphs were generated using Design Expert Software V8.0.7.1 (Stat-Ease Inc., Minneapolis, MN, USA). Multidimensional statistical analysis (cluster analysis) was performed with Minitab 16 Statistical software. The dendrogram was created with the ward linkage method and the squared Euclidian distance measure was also selected in the formation of five clusters based on the following responses: firmness, adhesiveness, GLVR, the cross over point (G′ = G″), stability, and Windex.

## Figures and Tables

**Table 1:** Numeric factors (composition of the samples) and response variables for emulsion oleogels formulated with sunflower oil oleogel structured with shellac wax.

Run	Numeric Factors		Responses					
	Wax (%) (m/m)	Water (%) (m/m)	Hardness (g)	Adhesive Force (g)	G_LVR_ (Pa)	Cross over Point G′ = G″	Stability	Whiteness Index
EM1	7	60	415.5 ± 43.13	127.5 ± 4.95	32,405 ± 3428.05	1028.28 ± 126.16	99.99 ± 0.00	78.50 ± 0.45
EM2	5	20	160 ± 15.55	68.5 ± 8.13	5946.3 ± 102.81	69.85 ± 9.25	99.93 ± 0.003	58.12 ± 1.36
EM3	7	40	600 ± 31.11	255 ± 2.82	40,513.5 ± 40,372.04	462.44 ± 39.6	99.98 ± 0.00	71.96 ± 0.16
EM4	7	20	560.5 ± 54.80	196.5 ± 16.61	35,938 ± 2804.39	1731.5 ± 54.16	99.96 ± 0.006	61.41 ± 0.17
EM5	3	40	134 ± 9.90	57.5 ± 3.53	5014.4 ± 409.41	54.33 ± 4.75	99.92 ± 0.004	64.65 ± 0.39
EM6	3	20	54 ± 1.41	27 ± 2.82	1064.2 ± 113.13	38 ± 3.92	99.88 ± 0.003	58.99 ± 0.33
EM7	5	40	342 ± 11.31	144.5 ± 14.85	22,300 ± 2406.99	166.8 ± 14.14	99.95 ± 0.01	70.69 ± 0.71
EM8	3	60	63 ± 2.82	28.5 ± 0.70	4264.2 ± 264.45	87.55 ± 7.28	99.95 ± 0.01	75.61 ± 0.30
EM9	5	60	301.5 ± 19.09	126 ± 4.24	15,928.5 ± 267.99	223.84 ± 19.82	99.98 ± 0.001	76.66 ± 0.20

**Table 2:** Analysis of variance for the responses resulted in the analysis of emulsion oleogels.

Source	Hardness (g)	Adhesive Force (g)	G′_LVR_ (Pa)	Cross over Point G′ = G″	Stability	Whiteness Index
Model F value	12.55	12.11	30.09	15.97	130.22	24.88
Model *p* value	0.0318	0.0333	0.0091	0.0226	0.0011	0.0041
A-Water % *p* value	0.5700	0.7633	0.0465	0.0710	0.0005	0.0004
B-Structurant % *p* value	0.0010	0.0058	0.0016	0.0037	0.0003	0.0605
AB	0.4519	0.4574	0.0777	0.1289	0.0138	0.9653
A^2^	0.1106	0.0728	0.0534	0.9035	0.5828	0.4254
B^2^	0.3547	0.1875	0.3203	0.7596	0.0916	0.8168
R^2^	0.9544	0.9528	0.9804	0.9638	0.9954	0.9688
Residual	0.2949	0.2454	0.2396	0.0007	4.449 × 10^−9^	0.0034
Cor total	6.46	5.20	12.55	0.0187	9.701 × 10^−7^	0.10

## Supplementary Materials

The following supporting information can be downloaded at: https://www.mdpi.com/article/10.3390/gels8110749/s1, Figure S1. Changes in the rheological properties of shellac wax based emulsion oleogel. (a) EM1 (G′  and G” ), EM3 (G′  and G” ) and EM4 (G′  and G” ); (b) EM2 (G′  and G″ ), EM5(G′  and G″ ), EM7 (G′  and G″ ) and EM10 (G′  and G″ ); (c) EM6 (G′  and G″ ), EM8 (G′  and G″ ), EM9 (G′  and G″ ) during amplitude sweep analysis, at 20 °C, frequency of 1 Hz with oscillatory stress varying from 0.01 to 100 %.
