# Supplementary material for: The Feasibility of Shellac Wax Emulsion Oleogels as Low-Fat Spreads Analyzed by Means of Multidimensional Statistical Analysis
Source: Gels. 2022 Nov 18;8(11):749. doi: 10.3390/gels8110749 (PMC9689311; doi:10.3390/gels8110749)
Supplement: Supplementary file 1 [file gels-08-00749-s001.zip › gels-2016038- supplemenatry materi.pdf]

gels

MDPI

Supplementary File

The Feasibility of Shellac Wax Emulsion Oleogels as Low-Fat Spreads Analyzed by Means of Multidimensional Statistical Analysis

Andreea Puşcaş [1,2] and Vlad Mureşan [1,2] *

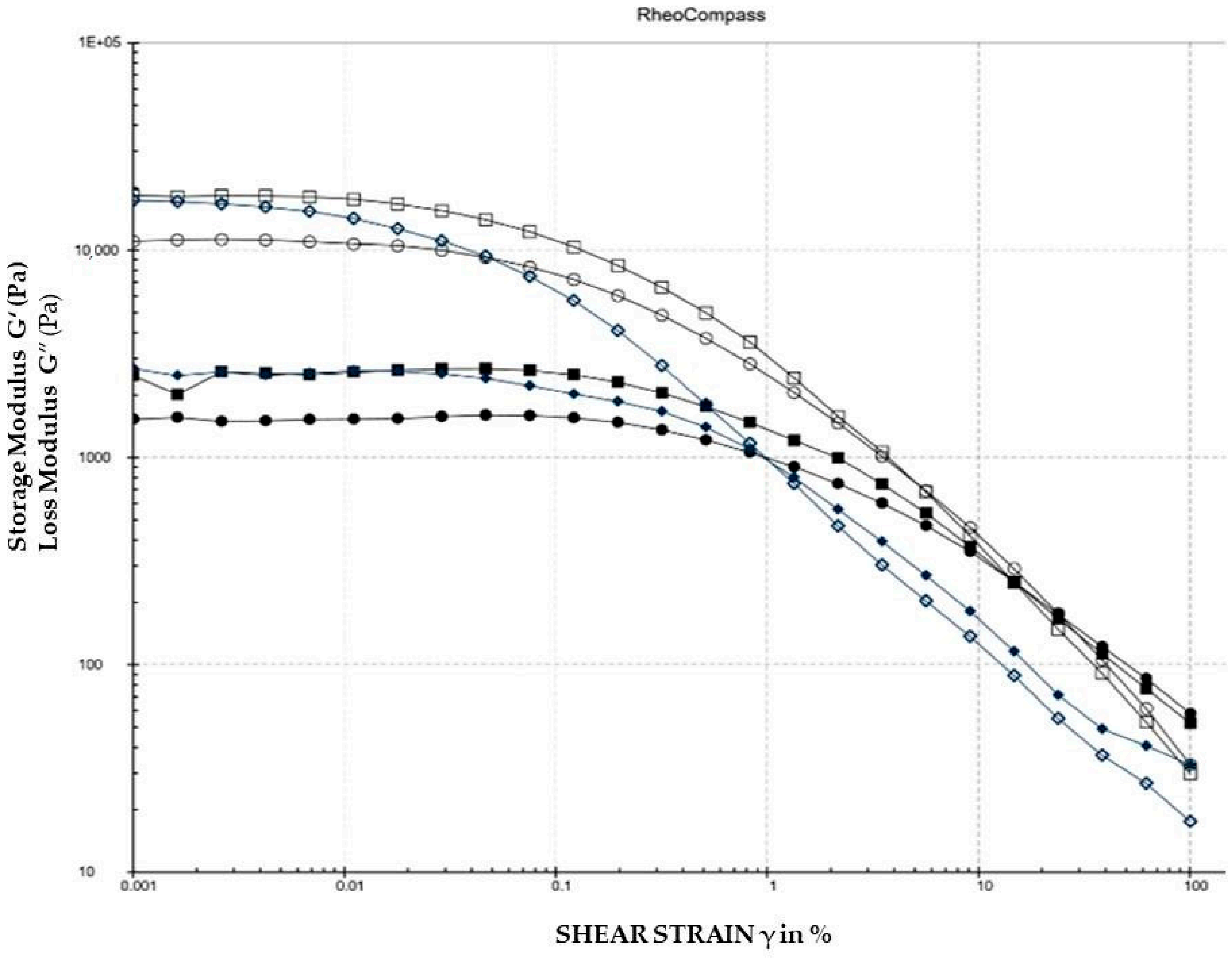

a)

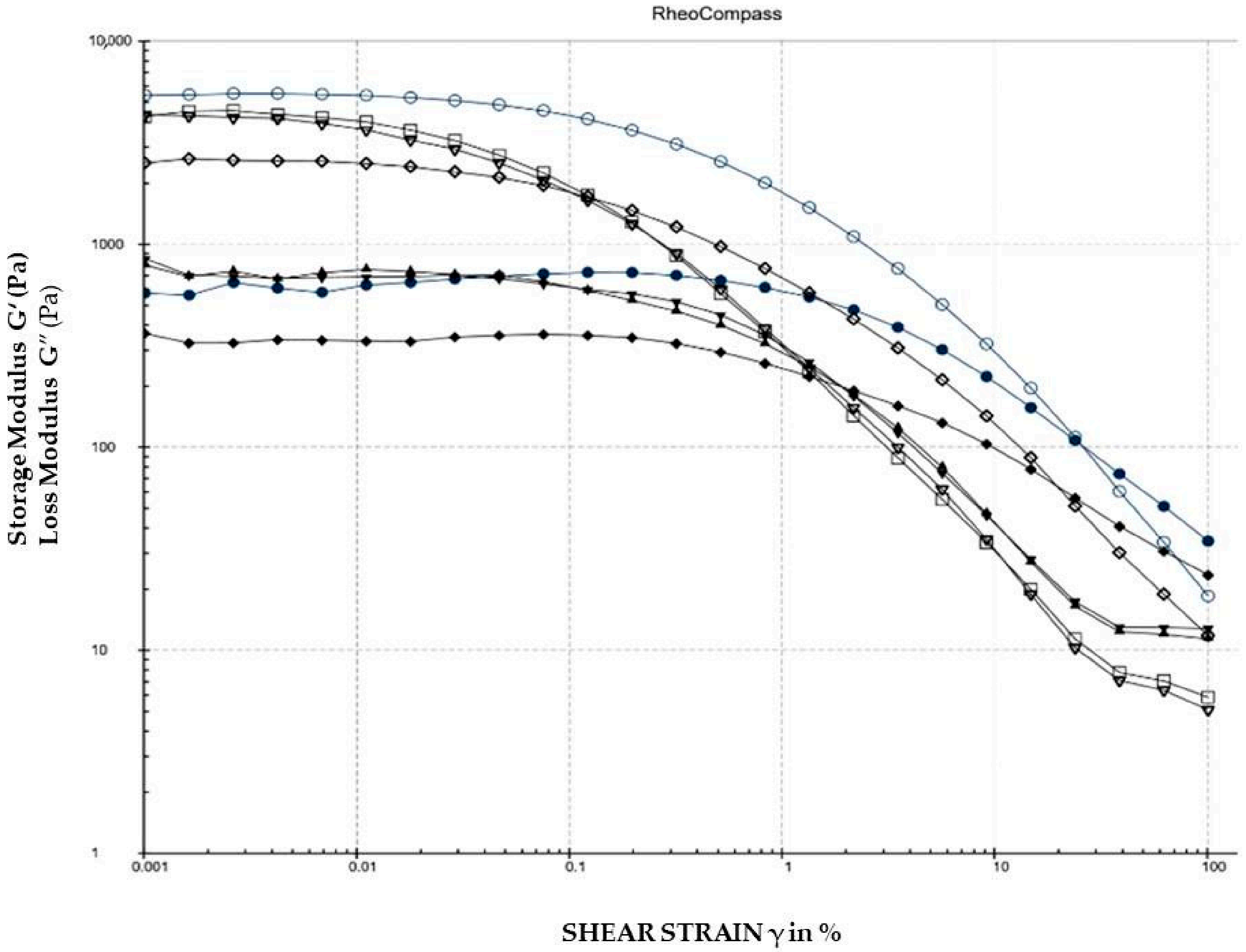
RheoCompass
10,000
1000
100
10
1
0.001
0.01
0.1
1
10
100
Storage Modulus G′ (Pa)
Loss Modulus G″ (Pa)
SHEAR STRAIN γ in %

b)

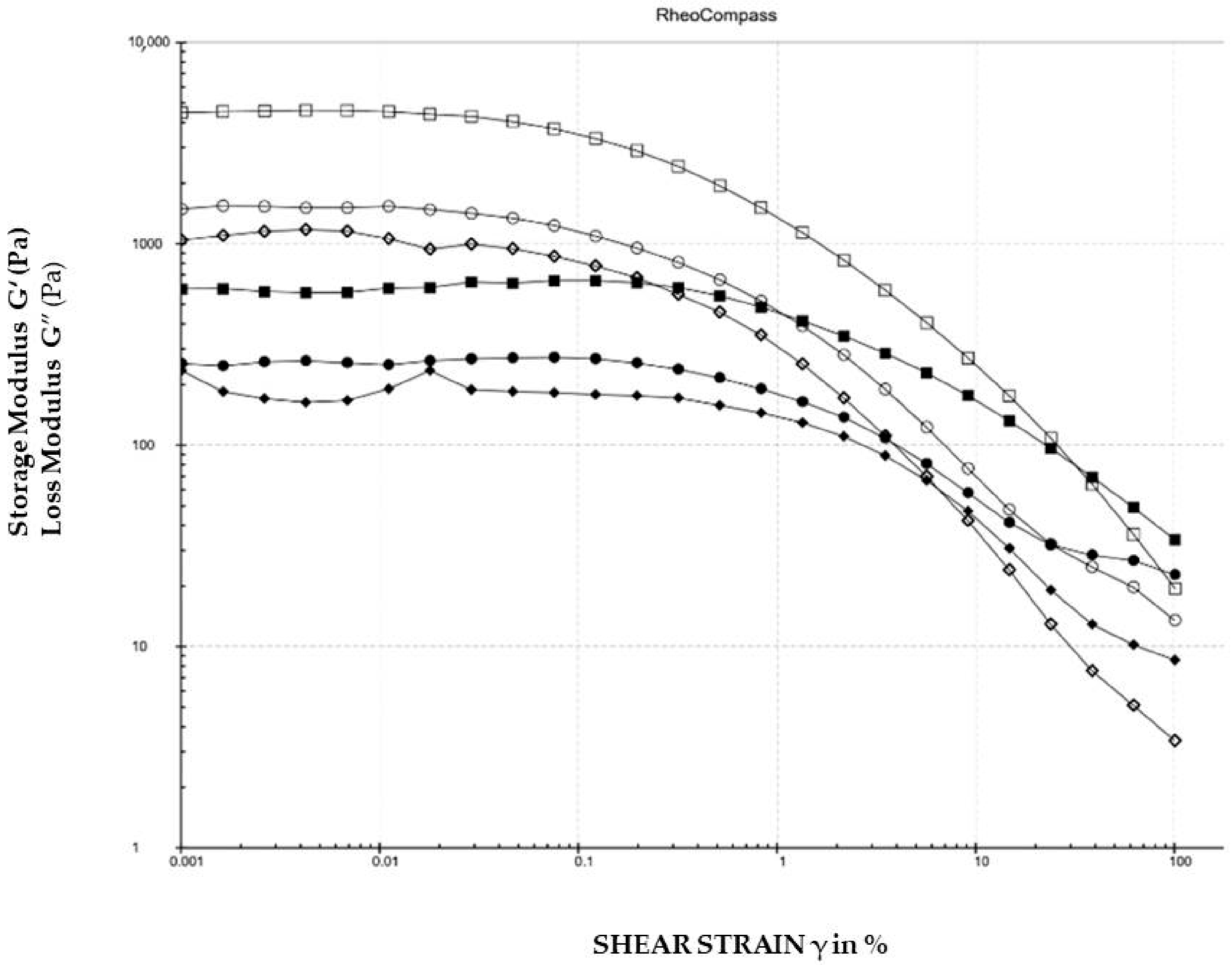

c)

Figure S1. Changes in the rheological properties of shellac wax based emulsion oleogel. a) EM1 (G′ and G″) , EM3 (G′ and G″) and EM4 (G′ and G″); b) EM2 (G′ and G″), EM5(G′ and G″) , EM7 (G′ and G″) and EM10 (G′ and G″); c) EM6 (G′ and G″), EM8 (G′ and G″), EM9 (G′ and G″) during amplitude sweep analysis, at 20°C, frequency of 1 Hz with oscillatory stress varying from 0.01 to 100 %.
